# Immune cells fold and damage fungal hyphae

**DOI:** 10.1073/pnas.2020484118

**Published:** 2021-04-05

**Authors:** Judith M. Bain, M. Fernanda Alonso, Delma S. Childers, Catriona A. Walls, Kevin Mackenzie, Arnab Pradhan, Leanne E. Lewis, Johanna Louw, Gabriela M. Avelar, Daniel E. Larcombe, Mihai G. Netea, Neil A. R. Gow, Gordon D. Brown, Lars P. Erwig, Alistair J. P. Brown

**Affiliations:** ^a^Aberdeen Fungal Group, Institute of Medical Sciences, Foresterhill, AB25 2ZD Aberdeen, United Kingdom;; ^b^Microscopy and Histology Facility, Institute of Medical Sciences, Foresterhill, AB25 2ZD Aberdeen, United Kingdom;; ^c^Medical Research Council Centre for Medical Mycology, University of Exeter, EX4 4QD Exeter, United Kingdom;; ^d^Department of Internal Medicine and Radboud Center for Infectious Diseases, Radboud University Medical Center, 6500HB Nijmegen, The Netherlands;; ^e^Department for Immunology and Metabolism, Life and Medical Sciences Institute, University of Bonn, 53115 Bonn, Germany;; ^f^Johnson-Johnson Innovation, Europe, Middle East and Africa Innovation Centre, London W1G 0BG, United Kingdom

**Keywords:** macrophages, fungal hyphae, mechanical force, podosomes, cytoskeleton

## Abstract

Innate immunity provides essential protection against life-threatening fungal infections. However, the outcomes of individual skirmishes between immune cells and fungal pathogens are not a foregone conclusion because some pathogens have evolved mechanisms to evade phagocytic recognition, engulfment, and killing. For example, *Candida albicans* can escape phagocytosis by activating cellular morphogenesis to form lengthy hyphae that are challenging to engulf. Through live imaging of *C. albicans*–macrophage interactions, we discovered that macrophages can counteract this by folding fungal hyphae. The folding of fungal hyphae is promoted by Dectin-1, β2-integrin, VASP, actin–myosin polymerization, and cell motility. Folding facilitates the complete engulfment of long hyphae in some cases and it inhibits hyphal growth, presumably tipping the balance toward successful fungal clearance.

## Significance

Macrophages protect against microbial infection, in part by engulfing and killing invading microbes. Fungal pathogens such as *Candida albicans* are known to evade phagocytic killing by forming hyphae that are physically challenging to engulf because of their length. We now find that macrophages can respond by folding the hyphae of *C. albicans* (and other fungal species). Hyphal folding implies that immune cells can continue to apply mechanical force after their cargo has been internalized. The involvement of Dectin-1, β2-integrin, and actin–myosin polymerization provides initial mechanistic insight. Folding damages hyphae, inhibits their growth, and facilitates their complete engulfment. Therefore, hyphal folding represents an additional weapon in the immune cell armory that presumably contributes to fungal clearance.

An estimated 1.5 million people succumb to a systemic fungal infection each year (1). Most of these individuals had HIV or had undergone a medical intervention that severely compromised their immunity. The innate immune system plays a key role in preventing fungal infection (2–4). The efficacy of these defenses depends on the outcome of individual interactions between innate immune cells and fungal pathogens.

Innate immune cells such as macrophages recognize fungal pathogens via pattern recognition receptors (PRRs) that interact with pathogen-associated molecular patterns (PAMPs), many of which lie at the fungal cell surface (5, 6). The formation of a phagocytic synapse between PRRs and PAMPs triggers the active engulfment of the pathogen and subsequent attempts to kill the fungal cell using a variety of mechanisms that include a toxic mix of reactive chemical species and antimicrobial peptides (2). Meanwhile, fungal pathogens attempt to evade immune recognition, phagocytosis, and killing through a range of strategies that include PAMP masking to reduce recognition (7, 8), robust stress responses to attenuate the potency of reactive oxygen and nitrogen species (9–11), the activation of pyroptosis to kill the immune cells (12–14), and in particular, cellular morphogenesis (15–18). *Candida albicans* activates morphogenetic programs to form hyphae that are challenging to phagocytose and clear, not least because of their extreme length (6, 15, 16). Hypha formation also provides a means of fungal escape from the macrophage (12, 13).

While examining dynamic interactions between macrophages and fungal cells, we observed that these immune cells can fold fungal hyphae. We reasoned that this fungal folding must involve the application of mechanical forces and that this folding contributes to fungal clearance. Therefore, we examined the involvement of the cytoskeletal network and PRRs in this phenomenon, providing initial clues as to how macrophages anchor a fungal hypha and achieve leverage to fold it.

## Results

### Macrophages Can Fold Fungal Hyphae.

**Fig. 1.**
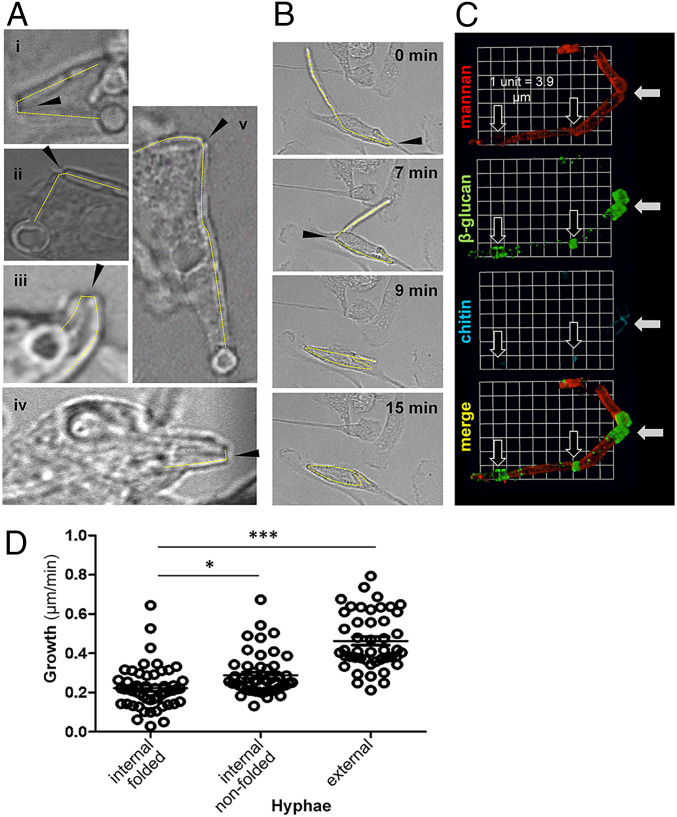
Physical manipulation and damage of *C. albicans* hyphae by macrophages. (*A*) Live *C. albicans* SC5314 cells were added to cultures of macrophages (multiplicity of infection 3:1): (i) BMDM, (ii) thio-macs, (iii) J774.1, (iv) RAW264.7, and (v) human monocyte–derived macrophages for up to 6 h of live-cell imaging. Selected movie frames show phagocytosed hyphae folding at sites indicated with arrows. Movies of fungal folding can be viewed in Movies S1–S5. (*B*) Selected frames from live-imaging movies showing longer fixed *C. albicans tup1*Δ hyphae being folded, thereby facilitating complete engulfment of the long particle. The arrows highlight two folding events on the same hypha. The corresponding movie can be viewed in Movie S6. (*C*) Live *C. albicans* SC5314 cells were allowed to interact with thio-macs for 4 h and then fixed. Exposure of cell wall mannan (ConA, red), β-glucan (Fc-Dectin-1, green), and chitin (WGA, blue) was examined. A representative three-dimensional (3-D) image reconstruction (3-D opacity) of a phagocytosed filament is shown, revealing fracture at the folded septal junction (white arrows). Partially or unfolded septal junctions are also highlighted (black arrows). (*D*) Human monocyte–derived macrophages were combined with live *C. albicans* SC5314 cells and their interactions imaged for 6 h. Movies were analyzed for phagocytic events and hyphal growth, and the Volocity line tool used to measure hyphal length and calculate growth rate (micrometers per minute). Hyphae were categorized according to whether they were internalized and folded, internalized but not folded, or not phagocytosed (external): *n* = 24 to 43 events per category. Statistical differences between groups were determined by ANOVA with Tukey’s multiple comparison test, post hoc; *P < 0.05, ***P < 0.001.

To monitor the dynamic interactions between fungal and innate immune cells we performed live imaging of macrophage cultures inoculated with live yeast cells of the wild-type *C. albicans* clinical isolate SC5314. Cell wall stains such as Calcofluor White perturb cell wall PAMPs (19, 20) and, thereby, immune interactions, and therefore we avoided such stains. As reported previously (15, 16), some yeast cells formed hyphae in response to the culture conditions or following phagocytosis, yielding morphologically diverse fungal populations. Interestingly, we found that some long hyphae were folded by the macrophages, often at fungal septal junctions, a known point of fragility (21) (Fig. 1*A* and Movies S1–S5). Innate immune cells have been reported to exert force at the cell surface in the act of phagocytosing *C. albicans* yeast cells (22). This suggests that immune cells continue to exert force following the phagocytic engulfment of fungal hyphae.

As mentioned, *C. albicans* cells display considerable morphological heterogeneity during live cell imaging of macrophage–fungus interactions. Therefore, we used a morphogenetically locked mutant to facilitate quantification of fungal folding. *C. albicans tup1*Δ cells lack Tup1, a transcriptional repressor of hyphal development (23). Consequently, they constitutively form long, nonaggregating pseudohyphal filaments. These *tup1*Δ filaments were phagocytosed, but direct comparisons of phagocytosis efficiencies for lengthy *tup1*Δ filaments and wild-type hyphae were not feasible because the wild-type hyphae aggregated to form clumps. Significantly, murine bone marrow–derived macrophages (BMDMs) folded these *tup1*Δ filaments, some of which were over 100 µm in length (Movie S6). Some filaments were folded multiple times (Fig. 1*B* and Movie S7). The ability of macrophages to fold fixed, nongrowing hyphae (Movies S6 and S7) clearly distinguishes this phenomenon from that of thigmotropism, which involves contact sensing and responses to mechanical forces during the polarized growth of hyphae (24–26).

To explore the generality of hyphal folding, we examined different types of macrophage. We observed folding of *C. albicans* hyphae by BMDMs, thioglycolate-elicited peritoneal macrophages (thio-macs), macrophage cell lines (J774.1 and RAW 264.7 cells), and human monocyte–derived macrophages. Furthermore, we found that BMDMs were capable of phagocytosing and folding hyphae of the evolutionarily divergent nonsporulating mold, *Mycelia sterilia* (Movie S8).

### Folding Damages Fungi.

We predicted that folding might damage the hypha. To test this, we examined points of fungal folding in more detail, observing indentations at septal fold sites (Fig. 1*A*) and, in some cases, breakage of fixed hyphae during folding (Movie S9). Imaging of cell wall components by immunofluorescence revealed significant disruption of cell wall architecture at these fracture sites. Chitin and β-glucan are normally located in the inner layer of the *C. albicans* cell wall (27, 28). However, staining of engulfed, live, folded hyphae with wheat germ agglutinin and Fc-Dectin-1 showed that chitin and β-glucan, respectively, became more exposed at fracture sites compared with unfolded septal junctions (Fig. 1*C*). Both chitin and β-glucan are immunomodulatory PAMPs (5, 29–31) that are normally covered by the mannan in the outer layer of the *C. albicans* cell wall (32). Therefore, folding disrupts the cell wall architecture of *C. albicans* hyphae to affect PAMP exposure.

Hyphal growth slows following internalization by macrophages (33). To test whether fungal folding exacerbates this growth inhibition, we performed live imaging of macrophages phagocytosing growing wild-type *C. albicans* cells. We measured the extension rates of individual hyphae over an hour-long period. Internalized hyphae, whether folded or not, were impaired in their growth compared with hyphae that had not been engulfed (Fig. 1*D*), as reported previously (33). Comparing internalized hyphae, the folded hyphae displayed significantly reduced extension rates compared with nonfolded hyphae. Therefore, mechanical folding retards fungal growth. In the context of antifungal immunity, this presumably promotes fungal clearance by reducing the escape of cargo from the macrophage.

### Folding Requires Actin and Myosin II.

**Fig. 2.**
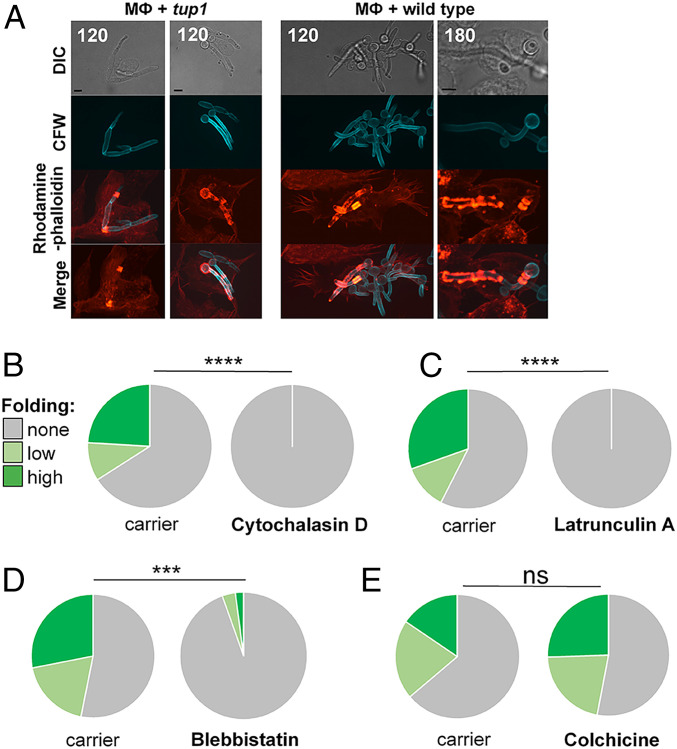
Role of cytoskeleton in hyphal folding. (*A*) BMDMs (MФ) were mixed with fixed *C. albicans tup1*Δ hyphae (*Left*) or live *C. albicans* SC5314 yeast cells (*Right*) stained with Calcofluor White (CFW, blue). Dynamic changes in actin localization were monitored by time-lapse fluorescence microscopy using rhodamine phalloidin staining (red). Dynamic patterns of actin patterns, including rings and punctate structures, were associated with *C. albicans–*containing phagosomes. Representative images at 120 or 180 min are shown. (*B*) Pretreatment of BMDMs with the actin inhibitor cytochalasin D (5 µM) blocks fungal folding, unlike the carrier, DMSO. Folding of preinternalized hyphae was quantified by scoring unfolded cells (gray), hyphae displaying moderate bending (obtuse angle; pale green), and hyphae displaying strong folding (acute angle; dark green). (*C*) The actin inhibitor latrunculin A (1 µM) inhibits fungal folding; carrier, DMSO. (*D*) The myosin II inhibitor blebbistatin (20 µM) inhibits fungal folding; carrier, DMSO. (*E*) Pretreatment with colchicine (10 µM), which inhibits microtubule polymerization, does not block folding: carrier, ethanol. The data for each inhibitor are from >100 uptake events from three independent experiments with BMDMs from different mice. Statistical comparisons were made using a χ^2^ test: ***P < 0.001; ****P < 0.0001; ns, not significant.

Actin forms dynamic structures around phagosomes containing *C. albicans* hyphae (16, 34). Therefore, we tested whether actin contributes to fungal folding. Using SiRActin (a silicon-rhodamine-based acting binding fluorophore) to monitor actin dynamics in macrophages, we observed that dynamic actin rings formed around *tup1*Δ hyphae, particularly around their septa (Movie S10). This was confirmed by rhodamine phalloidin staining of macrophages interacting with *tup1*Δ and wild-type cells (Fig. 2*A*). Some actin rings were located at hyphal folds (Fig. 2*A*).

We investigated whether actin functionality is required for hyphal folding. Actin inhibition blocks phagocytosis (35–37), and therefore to permit initial phagocytosis, macrophages were allowed to interact with *tup1*Δ filaments for 1.5 h before cytoskeletal inhibitors were added. *C. albicans*–macrophage dynamics were then imaged at 1 min intervals for a further 2 h, and the folding of internalized hyphae was categorized and quantified: none (no detectable bending or folding); moderate (creating a curved hypha or an obtuse angle), or high (generating an acute angle). Macrophages treated with the F-actin–binding reagent, cytochalasin D, were unable to fold their hyphal cargo, whereas those treated with carrier alone bent or folded around 35% of internalized hyphae (Fig. 2*B*). Similarly, macrophages exposed to the actin monomer–binding inhibitor latrunculin A were unable to fold hyphae (Fig. 2*C*). Therefore, actin functionality is essential for hyphal folding.

Myosin II contributes to the formation of actomyosin bundles capable of generating mechanical tension within mammalian cells (38–40). Therefore, we tested whether myosin II contributes to hyphal folding by pretreating macrophages with the inhibitor blebbistatin for 2 h before exposure to *tup1*Δ hyphae. Blebbistatin did not affect phagocytosis significantly (*SI Appendix*, Fig. S1*A*). However, blebbistatin-treated macrophages displayed significantly reduced folding compared with control macrophages treated with carrier alone (Fig. 2*D*). We conclude that macrophages also require myosin II to fold hyphae.

The mechanical delivery of cargo into target cells by cytotoxic T lymphocytes involves actin polarization and microtubule networks at the immunological synapse (41, 42). Therefore, we tested whether microtubules promote hyphal folding by treating macrophages with colchicine, a microtubule inhibitor. The phagocytosis of *tup1*Δ hyphae was unaffected by colchicine (*SI Appendix*, Fig. S1*B*), and significantly, fungal folding was not impaired (Fig. 2*E*), suggesting that microtubules are not required for hyphal folding.

### PAMP–PRR Synapses Promote Folding.

**Fig. 3.**
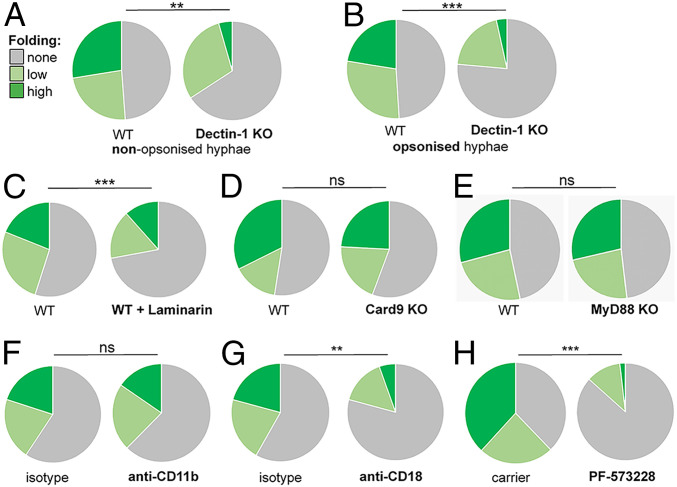
PAMP–PRR interactions but not Syk1-CARD9 signaling promote hyphal folding. (*A*) Inactivation of Dectin-1 blocks folding of nonopsonized *C. albicans tup1*Δ hyphae by BMDMs: wild-type (WT) BMDMs; Dectin-1 KO (*n* = 44), BMDMs from Dectin-1^−/−^ mice (*n* = 111); unfolded cells (gray), hyphae displaying moderate bending (obtuse angle; pale green), and hyphae displaying (acute angle; dark green). (*B*) Folding of opsonized *C. albicans tup1*Δ hyphae is attenuated in BMDMs from Dectin-1^−/−^ mice (*n* = 98) compared with WT BMDMs (*n* = 209). (*C*) The Dectin-1 blocking agent, laminarin, reduces folding of fixed *tup1*Δ hyphae by BMDMs from WT mice: *n* = 305 (+ laminarin); *n* = 337 (− laminarin). (*D*) Inactivation of CARD9 does not inhibit folding: WT (*n* = 59); CARD9 KO (*n* = 81). (*E*) Inactivation of MyD88 does not affect folding: MyD88 KO (*n* = 115) compared with WT (*n* = 113). (*F*) Pretreatment of BMDMs with antibodies against the glucan binding domain of CD11b (anti-CD11b) did not affect folding of fixed *tup1*Δ hyphae compared with treatment with isotype controls: *n* = 122 (anti-CD11b) and *n* = 107 (isotype control). (*G*) In contrast, pretreatment of BMDMs with antibodies against CD18 (anti-CD18) reduced folding of the *tup1*Δ hyphae compared with the isotype controls: *n* = 144 (anti-CD18) and *n* = 145 (isotype). (*H*) The Focal Adhesion Kinase inhibitor, PF573288 (10 µM), attenuated folding of the *tup1*Δ hyphae; *n* = 60 (PF573288); *n* = 87 (DMSO carrier). The data for each experiment are from three independent analyses with BMDMs from different mice. Statistical comparisons were made using a χ^2^ test: **P < 0.01; ***P < 0.001; ****P < 0.0001; ns, not significant.

How does a macrophage grasp a fungal hypha in order to fold it? We reasoned that PAMP–PRR synapses provide a potential means of physically connecting a hypha to the cytoskeleton across the phagosomal membrane. The C-type lectin, Dectin-1, plays a key role in fungal recognition and phagocytosis by interacting with β-glucan exposed at the fungal cell surface (5, 29, 30). Furthermore, Dectin-1 has been reported to potentiate the formation of intense actin cuffs around phagosomes that are attempting to engulf fungal hyphae (33). Therefore, we tested whether this PRR also plays a role in hyphal folding by comparing macrophages from Dectin-1^−/−^ knockout and wild-type mice. Our live imaging confirmed previous reports (43, 44) that Dectin-1 inactivation reduces the ability of macrophages to phagocytose *C. albicans* cells. In our hands, the Dectin-1^−/−^ macrophages phagocytosed fixed *tup1*Δ hyphae at 28% of the wild-type rate. Those Dectin-1^−/−^ macrophages that did successfully engulf a hypha displayed significantly reduced frequencies of hyphal folding (Fig. 3*A*). Furthermore, this folding was less severe (i.e., fewer hypha were folded at an acute angle).

We repeated the experiment with serum-opsonized hyphae, which enhanced phagocytosis by the Dectin-1^−/−^ macrophages to 47% of the wild-type rate. Again, Dectin-1^−/−^ macrophages were less able to fold their hyphal cargo than control wild-type macrophages (Fig. 3*B*). We then tested the effects of pretreatment with laminarin, a soluble beta-glucan that blocks Dectin-1 (45, 46). Laminarin pretreatment reduced but did not completely block the uptake of the *C. albicans* cells by wild-type macrophages, as expected (45, 46). Analyses of those hyphae that were phagocytosed revealed that laminarin inhibited hyphal folding (Fig. 3*C*). These data indicate that the engagement of fungal β-glucan by Dectin-1 enhances hyphal folding within the macrophage. This reinforces the view that this PAMP–PRR interaction continues to promote macrophage functionality after phagocytosis (47, 48).

We probed Dectin-1 signaling by assessing the contribution of caspase recruitment domain–containing protein 9 (CARD9), which functions downstream in this pathway (49, 50). Our comparisons of BMDMs from CARD9^−/−^ knockout and wild-type mice revealed no loss of hyphal folding by the CARD9^−/−^ macrophages (Fig. 3*D*). Also, Dectin-1 inhibition did not block folding completely (Fig. 3 *A*–*C*). Therefore, other PAMP–PRR interactions may contribute to the phenomenon. We extended our analysis to include Toll-like receptor (TLR) signaling by examining myeloid differentiation primary response 88 (MyD88) (51). BMDMs from MyD88^−/−^ mice displayed a similar hyphal folding capacity to their wild-type controls (Fig. 3*E*). We conclude that while PAMP–PRR interactions are required for fungal folding (Fig. 3 *A*–*C*), downstream PRR signaling via CARD9 or MyD88 is not (Fig. 3 *D* and *E*).

We examined the role of integrin in folding because it provides an additional means by which immune cells can engage an external target across a membrane (52, 53). Also, the β2-integrin, complement receptor 3 (CR3), is another type of β-glucan receptor (54) that promotes macrophage responses in collaboration with Dectin-1 (55). CD11b and CD18 are subunits of CR3. Therefore, we pretreated macrophages with M1/70 anti-CD11b antibody to block the β-glucan–specific domain of CD11b, and then quantified their ability to fold *tup1*Δ hyphae. Blocking CD11b did not disrupt hyphal folding (Fig. 3*F*). However, antibody-mediated inhibition of CD18, which compromises the functionality of all four members of the β2-integrin group, significantly reduced folding (Fig. 3*G*) without affecting phagocytosis (*SI Appendix*, Fig. S1*C*). To further assess the role of this PRR in folding, we blocked the downstream signaling component, focal adhesion kinase (FAK), using the inhibitor PF-573228 (56). PF-573228–treated macrophages phagocytosed *tup1*Δ hyphae as efficiently as the carrier-only control cells (*SI Appendix*, Fig. S1*D*) but were less able to fold them (Fig. 3*H*). Therefore, Dectin-1 and β2-integrin may collaborate to promote folding.

### Podosomes and Folding.

How do phagosomal synapses promote folding? Podosomes have been associated with functions such as the mechanosensing of phagocytic targets and local topological properties during tissue migration (57, 58), and podosomes have been implicated in the early stages of phagosome formation (59). Furthermore, integrin promotes the formation of podosome-like structures during phagocytosis (58, 59), and it plays a role in hyphal folding (Fig. 3*G*).

**Fig. 4.**
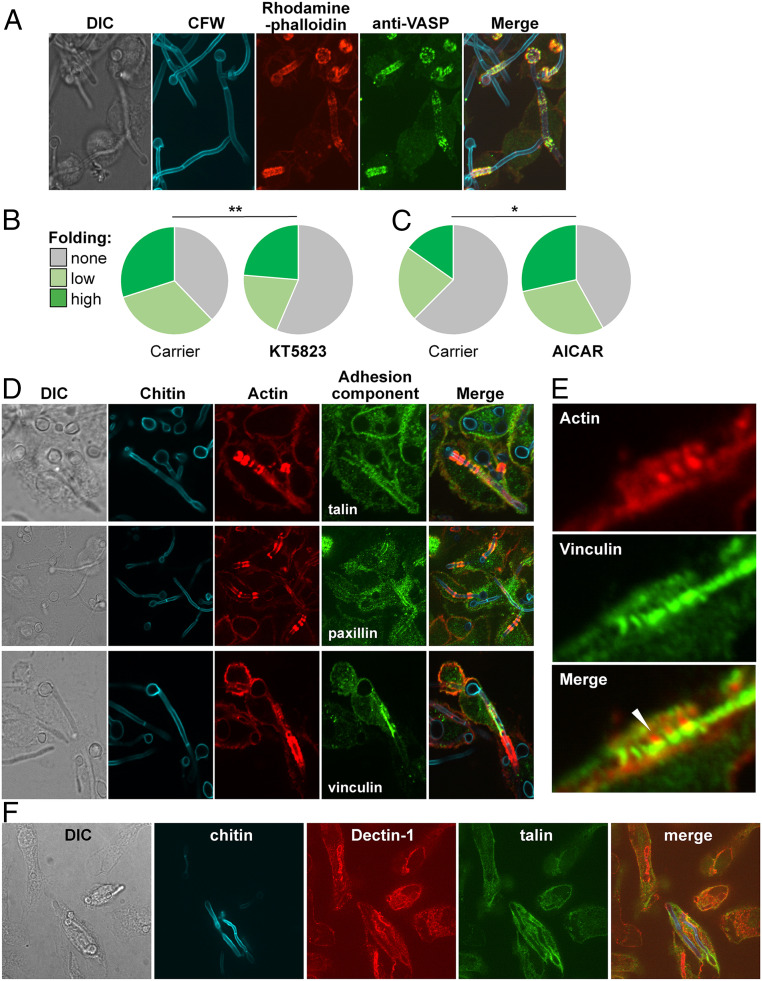
Podosome components associated with phagocytosed hyphae. (*A*) VASP (anti-VASP antibody, green) colocalizes with actin (rhodamine phalloidin, red) on BMDM phagosomes containing live *C. albicans* SC5413 (Calcofluor White; CFW, blue). Cells were fixed after 4 h of *C. albicans*–BMDM interactions and subjected to phase differential interference contrast (DIC) and fluorescence microscopy. (*B*) Pretreating BMDMs with KT5823 (0.1 µM), which reduces phosphoVASP levels, decreases their folding of fixed *tup1*Δ hyphae (*n* = 160) compared with the ethyl acetate carrier control (*n* = 186): unfolded cells (gray); hyphae displaying moderate bending (obtuse angle; pale green); hyphae displaying (acute angle; dark green). (*C*) Preincubating with AICAR (0.3 mM), which increases phosphoVASP levels, enhances the ability of BMDMs to fold *tup1*Δ hyphae (*n* = 145) compared with the H_2_O_2_ carrier control (*n* = 139). The data for each experiment are from three independent analyses with BMDMs from different mice. Statistical comparisons were made using a χ^2^ test: *P < 0.05; **P < 0.01. (*D*) Podosomal components localize with actin rings around phagosomes containing live *C. albicans* SC5314 hyphae. Three independent examples of this colocalization are shown: phase DIC microscopy, fungal chitin (CFW, blue), BMDM actin (rhodamine phalloidin, red), talin (anti-talin antibody, green; *Top*), paxillin (anti-paxillin antibody, green; *Middle*), and vinculin (anti-vinculin antibody, green; *Lower*). (*E*) A close-up showing juxtaposition of actin (rhodamine phalloidin, red) and vinculin (anti-vinculin antibody, green) on a BMDM phagosome containing a *C. albicans* SC5314 hypha. (*F*) Colocalization of Dectin-1 (anti-Dectin-1, red) and talin (anti-talin antibody, green) on BMDM phagosomes containing *C. albicans* SC5314 hyphae (chitin stained with CFW, blue).

Vasodilator-stimulated phosphoprotein (VASP) regulates the formation of contractile actomyosin bundles to generate mechanical tension in nonmuscle cells (60) and is essential for podosome formation (61). Interestingly, VASP colocalized with dynamic actin structures surrounding phagosomes that contain internalized *C. albicans* hyphae (Fig. 4*A*). VASP is regulated by adenosine monophosphate–activated protein kinase (AMPK) (62). Macrophages treated with the AMPK inhibitor KT5823 (63–65) were less able to fold hyphae than control cells (Fig. 4*B*). Conversely, macrophages exposed to the AMPK activator 5-Aminoimidazole-4-carboxamide ribonucleotide (AICAR) (63–65) displayed enhanced folding compared with control macrophages (Fig. 4*C*). Taken together, these observations suggest that VASP activation promotes the hyphal folding.

**Fig. 5.**
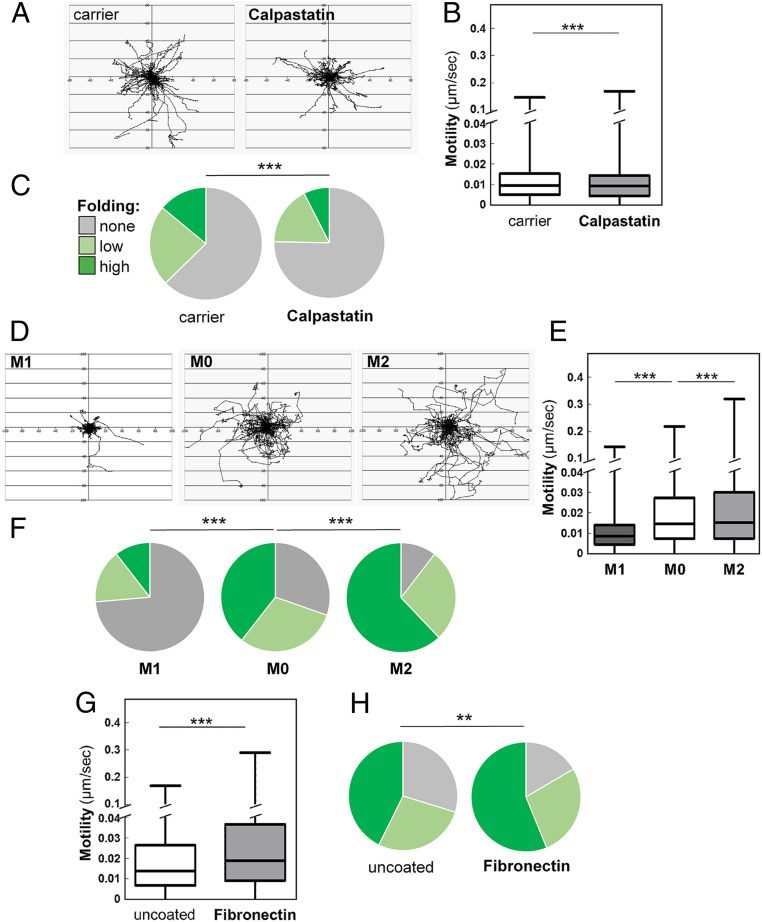
Influencing podosomal functionality or cell motility affects fungal folding. (*A*) Pretreatment with calpastatin (50 µM), which inhibits podosome turnover and function, decreases BMDMs motility as determined by tracking the movement of individual macrophages for 1 h: *n* = 30 per condition. (*B*) Quantification of BMDM motility based on the tracking shown in (*A*). (*C*) Folding of fixed *C. albicans tup1*Δ hyphae was impaired in calpastatin-treated BMDMs (50 μM, *n* = 193), compared with the carrier alone (H_2_O, *n* = 186): unfolded cells (gray); hyphae displaying moderate bending (obtuse angle; pale green); hyphae displaying (acute angle; dark green). (*D*) Human monocyte–derived were either untreated (M0), activated with IFN-γ and lipopolysaccharide (M1), or with IL-4 (M2) and then analyzed. Individual cells were tracked: *n* = 30. (*E*) The motility of these cells was then quantified. **P < 0.01; ***P < 0.001. (*F*) The ability of these cells to fold *C. albicans tup1*Δ hyphae was also quantified: M0 (*n* = 152 uptake events); M1 (*n* = 104); M2 (*n* = 147). (*G*) Human monocyte–derived macrophages were plated on standard or fibronectin-coated imaging dishes, combined with fixed *tup1*Δ hyphae, and imaged. Their motility was assayed by tracking individual cells (*n* = 30 for each condition). (*H*) Their ability to fold the *tup1*Δ hyphae was also quantified: *n* = 278, uncoated plates; *n* = 328, fibronectin-coated plates. The data for each experiment are from three independent analyses with macrophages from different mice or human donors. Statistical comparisons of folding were made using a χ^2^ test, and statistical analyses of the motility datasets used the unpaired *t* test (*B* and *G*) or ANOVA (GraphPad Prism).

We investigated whether other components of focal adhesions and podosomes are associated with hypha-containing phagosomes by immunofluorescence microscopy. Talin, paxillin, and vinculin were observed at macrophage periphery in focal adhesions and podosomes, as described previously (66, 67). Significantly, we also observed these proteins internally, surrounding phagosomes containing *C. albicans* hyphae (Fig. 4*D* and *SI Appendix*, Fig. S2), closely associated with actin bundles surrounding internalized hyphae (Fig. 4*E*). We also observed Dectin-1 on hypha-containing phagosomes (Fig. 4*F* and *SI Appendix*, Fig. S3). Calpastatin inhibits calpain-mediated turnover of podosomes (68). As expected (68), calpastatin reduced macrophage motility slightly (Fig. 5 *A* and *B*). Significantly, calpastatin also inhibited hyphal folding (Fig. 5*C*). Taken together, these observations suggest that podosome-like structures may contribute to hyphal folding.

### Migratory Activity and Folding Capacity.

Calpastatin affects cell motility as well as hyphal folding (Fig. 5 *A* and *B*). Also, perturbation of cytoskeletal functionality attenuates folding (Fig. 2). Therefore, we investigated the relationship between cell motility and folding by interfering with motility in other ways.

The migratory capacity of macrophages depends on their activation status (69). Therefore, human monocyte–derived macrophages were used to generate M0, M1, or M2 cells. These three macrophage subsets were then exposed to *tup1*Δ hyphae, and their behaviors were then examined by live-cell imaging. The M1 cells were less motile than the M0 controls and displayed less folding (Fig. 5 *D*–*F*). Conversely, the M2 macrophages displayed increased motility and enhanced folding.

Extracellular matrix components enhance the motility of migratory cells (70–72). Therefore, human monocyte–derived macrophages were plated onto plastic or plastic coated with fibronectin. Macrophages adhered to these substrata were fed *tup1*Δ hyphae, and their folding capacity and motility examined by live imaging. The fibronectin-coated surface increased the folding capacity of BMDMs as well as their motility (Fig. 5 *G* and *H*).

These data suggest that hyphal folding is related to the migratory capacity of macrophages. However, folding is not entirely governed by motility because Dectin-1^−/−^ macrophages displayed slightly increased motilities (*SI Appendix*, Fig. S4) but reduced hyphal folding (Fig. 3 *A* and *B*).

## Discussion

Lymphocytes invoke actin polarization and microtubule networks during their mechanical delivery of cargo to target cells (41, 73), and it is well known that phagocytes employ actin foci during their engulfment of targets (16, 22, 57, 59). Our demonstration that macrophages can fold fungal hyphae suggests that they are able to exert physical force upon internalized targets, postphagocytosis. This folding appears to be a general phenomenon executed by a range of phagocytic cell types and on evolutionarily diverse fungal targets.

Our data provide initial insights into the mechanics of hyphal folding. Dectin-1 and β2-integrin individually promote but are not essential for folding, which suggests that these PRRs may collaborate or that other factors may contribute to the process. Other C-type lectin receptors that recognize mannans or chitin may play a role in this process, such as Dectin-2, mannose receptor, or Mincle, for example. Folding is not dependent on downstream CARD9 signaling from C-type lectin receptors or MyD88 signaling from TLRs. This might imply that the formation of PAMP–PRR complexes, rather than downstream signaling via these pathways, may be critical for hyphal folding. These complexes may anchor the hypha to the folding apparatus, which are located in the phagosome and cytoplasm, respectively.

The folding apparatus appears to require dynamic cytoskeletal remodelling of actin and myosin II, rather than microtubule functionality. Also, the folding capacity of macrophages appears to be related to their motility, suggesting that hyphal folding may exploit forces generated by the cytoskeletal network as it drives cell migration. The involvement of β2-integrin and VASP signaling, together with the colocalization of the adhesion components talin, paxillin, and vinculin at the surface of hypha-containing phagosomes, is consistent with the idea that podosome-like structures may link the PAMP–PRR anchor to the cytoskeleton, thereby permitting the transmission of this force from the cytoskeleton to the fungal hypha.

Hyphal folding damages the hypha and attenuates its growth. Therefore, we suggest that hyphal folding represents an additional weapon in the armory of the phagocyte, alongside reactive chemical species, antimicrobial peptides, and enzymes, for example. When combined with these other weapons, hyphal folding might help to tip the balance in favor of the phagocyte during antifungal immunity. The phagocytosis of hyphae is not dependent on folding. Nevertheless, by assisting the complete uptake of some long filaments, hyphal folding presumably reduces the likelihood of inappropriate immune activation via “frustrated” phagocytosis by macrophages (33), for example. While we have focused on the folding of fungal hyphae, we note that phagocytes may exert analogous forces upon other forms of cargo to promote their clearance.

## Materials and Methods

### Fungal Strains.

The *C. albicans* clinical isolate SC5314 (74) and the *C. albicans tup1*Δ null mutant CRC003 (23) were used throughout. These were grown in YPD (2% dextrose, 2% mycological peptone, and 1% yeast extract) at 30 °C overnight before examining their interactions with macrophages in Roswell Park Memorial Institute (RPMI)-1640 medium. For some experiments, fungal cells were fixed in 50 mM Thimerosal (Sigma-Aldrich) overnight and then washed thoroughly in phosphate-buffered saline (PBS).

### Phagocyte Preparation.

J774.1 and RAW264.7 macrophages were cultured at 37 °C with 5% CO_2_ using Dulbecco’s Modified Eagle Medium (DMEM) supplemented with 10% (volume/volume) heat-inactivated fetal calf serum (FCS: Sigma) and 200 U/mL penicillin/streptomycin (Sigma). Thioglycolate-elicited peritoneal macrophages [thio-macs (16)] and BMDMs (75) were generated as described previously. For some experiments, BMDMs were prepared from knockout mice lacking CARD9, Dectin-1, or MyD88 (43, 49, 76). BMDMs were treated at the following final concentrations in the specified carriers: 5 μM cytochalasin D in dimethyl sulfoxide (DMSO), 1 μM latruncilin A in DMSO, 20 μM blebbistatin in DMSO, 10 μM colchicine in EtOH, 100 μg/mL laminarin in RPMI, anti-Cd11b or isotype (abcam 8878) added at 33 μg/mL, anti-CD18 or isotype (abcam 119830) added at 33 μg/mL, 10 μM PF573228 in DMSO, 1 μM KT5823 in ethyl acetate, 0.3 mM AICAR in H_2_O, and 50 μM calpastatin in H_2_O.

Human monocyte–derived macrophages were prepared from the blood of healthy volunteers using density centrifugation followed by magnetic-activated cell sorting of CD14+ populations (Miltenyi Biotech) and cultured in DMEM containing 10% autologous human serum (77). For experiments with differentially activated monocytes, cells were left untreated until day 7 to generate M0 cells. M1 cells were generated by treating M0 cells with 50 ng/mL IFN-γ on day 5, then with 10 ng/mL lipopolysaccharide on day 6. M2 cells were generated by exposing M0 cells to 10 ng/mL IL-4 on day 5 for 48 h. Monocytes were analyzed on day 7. The monocytes were imaged on fibronectin-coated or standard tissue culture plastic. Fibronectin coating was performed by adding 5 µg/cm^2^ fibronectin in water to imaging wells and allowing evaporation.

### Visualization of Cell Components.

To visualize exposure of fungal cell wall components at fracture points of phagocytosed and folded filaments, exposed mannan was stained with concanavalin A (ConA) conjugated to Alexa Fluor 594 (Invitrogen), exposed chitin was stained with wheat germ agglutinin (WGA) conjugated to Alexa Fluor 350, exposed β-glucan was stained with Fc-Dectin-1 (78), and then goat F(ab′)2 antihuman IgG conjugated to Alexa Fluor 488. After coincubation of thio-macs and SC5314 for 4 h, macrophages were lysed with dH_2_O for 30 min, and fungal cells were fixed in 4% paraformaldehyde for 45 min and then washed twice with PBS and once with staining buffer (PBS containing 1% FCS and 0.5 mM ethylenediaminetetraacetic acid [EDTA]). Staining was performed in two sequential steps. First, cells were incubated with 1.0 µg/mL Fc-Dectin-1 in staining buffer for 45 min on ice. Then cells were incubated with 50 µg/mL WGA, 25 µg/mL ConA, and 1:200 Gt F(ab’) antihuman IgG in staining buffer for 30 min on ice. Control cells were only stained with the secondary antibody.

BMDM cellular components were visualized following phagocytosis of live SC5314 or fixed *tup1*Δ filaments after specified time intervals, as required. Cells were fixed with 2% paraformaldehyde for 10 min and then washed thrice with PBS. Fungal chitin was visualized by Calcofluor White staining (10 mg ⋅ mL^−1^ in dH_2_O for 20 min, or overnight if combined with primary antibody). Fixed actin was stained using rhodamine phalloidin (Invitrogen) at 1:200 for 1 h, or overnight if combined with primary antibody. Immunofluorescent staining was performed on fixed cells following permeabilization with 0.2% Triton-X 100. Key BMDM components were visualized by staining with 1:400 anti-VASP (Cell Signal Technologies 3132), 1:100 anti-talin (Abcam 71333), 1:100 anti-paxillin (Abcam 32084), or 1:250 anti-vinculin (Abcam 129002) in antibody buffer (PBS containing 1% FCS, 0.5 mM EDTA in PBS for VASP, or 0.5% saponin and 5% goat serum for talin, paxillin, and vinculin) overnight at 4 °C, then washed. Secondary antibody goat anti-rabbit Alexa Fluor 488 was applied at 1:200 in antibody buffer for 1 h at room temperature. Dectin-1 was visualized in fixed cells using 10 μg/mL of anti-Dectin-1 antibody 7G7 in 0.25% saponin, 1% bovine serum albumin, and 1% goat serum (overnight, chilled). Goat anti-rat Alexa Fluor 594 secondary antibody was applied at 1:200 for 1 h. Live actin was stained using SiRActin (Spirochrome) at 200 nM for 4 h prior to imaging.

### Live Imaging of Fungal-Phagocyte Interactions.

The folding of internalized fungal cargo was quantified first by performing live imaging using a PerkinElmer UltraVIEW VoX Spinning Disk microscope (Nikon ×20/0.75 Plan Apo VC objective). Movies were acquired using Volocity software 6.4 (PerkinElmer), collecting images at 2 min intervals for up to 6 h for wild-type cells and for up to 4 h for *tup1*Δ cells. Some data were collected using a Zeiss Celldiscoverer 7, capturing images with a ×20/0.95 Plan Apo objective. For macrophages that had phagocytosed a fungal filament, the folding of the hypha was categorised as “none” (no detectable bending or folding); “low” (creating a curved hypha or an obtuse angle), or “high” (generating an acute angle). Experiments were performed independently on multiple occasions using innate immune cells from at least three mice or human donors. Statistical analyses were performed using the χ^2^ test in GraphPad Prism.

The motility of innate immune cells was measured first by selecting random macrophages at frame 1, including non/phagocytosing and non/folding macrophages. Volocity manual tracking was performed on the selected macrophages by marking the central point of each macrophage with each advancing frame for the first 60 min of the movie. Individual track steps were combined (per condition) to determine mean velocity (μm/sec): n = 30 cells for each condition; n = 3 independent experiments. Statistical comparisons of two conditions were performed using an unpaired *t* test or χ^2^ test, as specified, and using ANOVA for three or more conditions (GraphPad Prism).

Cellular localization was examined by capturing Z stack images (0.3 µm step size) using a PerkinElmer UltraVIEW Vox spinning disk microscope using a Nikon Plan Apo VC ×60/1.4 oil objective.

Growth of *C. albicans* hyphae during phagocytosis and folding was determined by measuring hyphal extension rates with the line tool in Volocity.

### Ethics Statement.

Blood from healthy volunteers was collected with the informed consent of these donors and according to local guidelines and regulations. Our full study protocol was approved by the College Ethics Review Board of the University of Aberdeen (2016/8/1300).

Thio-macs were prepared from 10 to 14 wk old C57BL/6 female mice and BMDMs from 8 wk old male C57BL/6, CARD9^−/−^, Dectin-1^−/−^, or MyD88^−/−^ mice. These mice were selected randomly, bred in house, and housed in stock cages under specific pathogen-free conditions. They underwent no surgical procedures prior to culling by cervical dislocation. All animal experimentation was approved by the UK Home Office and by the University of Aberdeen Animal Welfare and Ethical Review Body.

## Supplementary Material

Supplementary File

Supplementary File

Supplementary File

Supplementary File

Supplementary File

Supplementary File

Supplementary File

Supplementary File

Supplementary File

Supplementary File

Supplementary File

Supplementary File

## Data Availability

All study data are included in the article and/or supporting information.
