# Supplementary material for: Immune cells fold and damage fungal hyphae
Source: Proc Natl Acad Sci U S A. 2021 Apr 5;118(15):e2020484118. doi: 10.1073/pnas.2020484118 (PMC8053999; doi:10.1073/pnas.2020484118)
Supplement: Supplementary File [file pnas.2020484118.sapp.pdf]

PNAS
www.pnas.org

Supplementary Information for

Immune cells fold and damage fungal hyphae.

Judith M. Bain, Fernanda Alonso, Delma S. Childers, Catriona A. Walls, Kevin Mackenzie, Arnab Pradhan, Leanne E. Lewis, Johanna Louw, Gabriela M. Avelar, Daniel E. Larcombe, Mihai G. Netea, Neil A.R. Gow, Gordon D. Brown, Lars P. Erwig and Alistair J.P. Brown

Communicating Author:
Alistair J.P. Brown
Email: a.j.p.brown@exeter.ac.uk

This PDF file includes:

Figures S1 to S4
Legends for Movies S1 to S11

Other supplementary materials for this manuscript include the following:

Movies S1 to S11

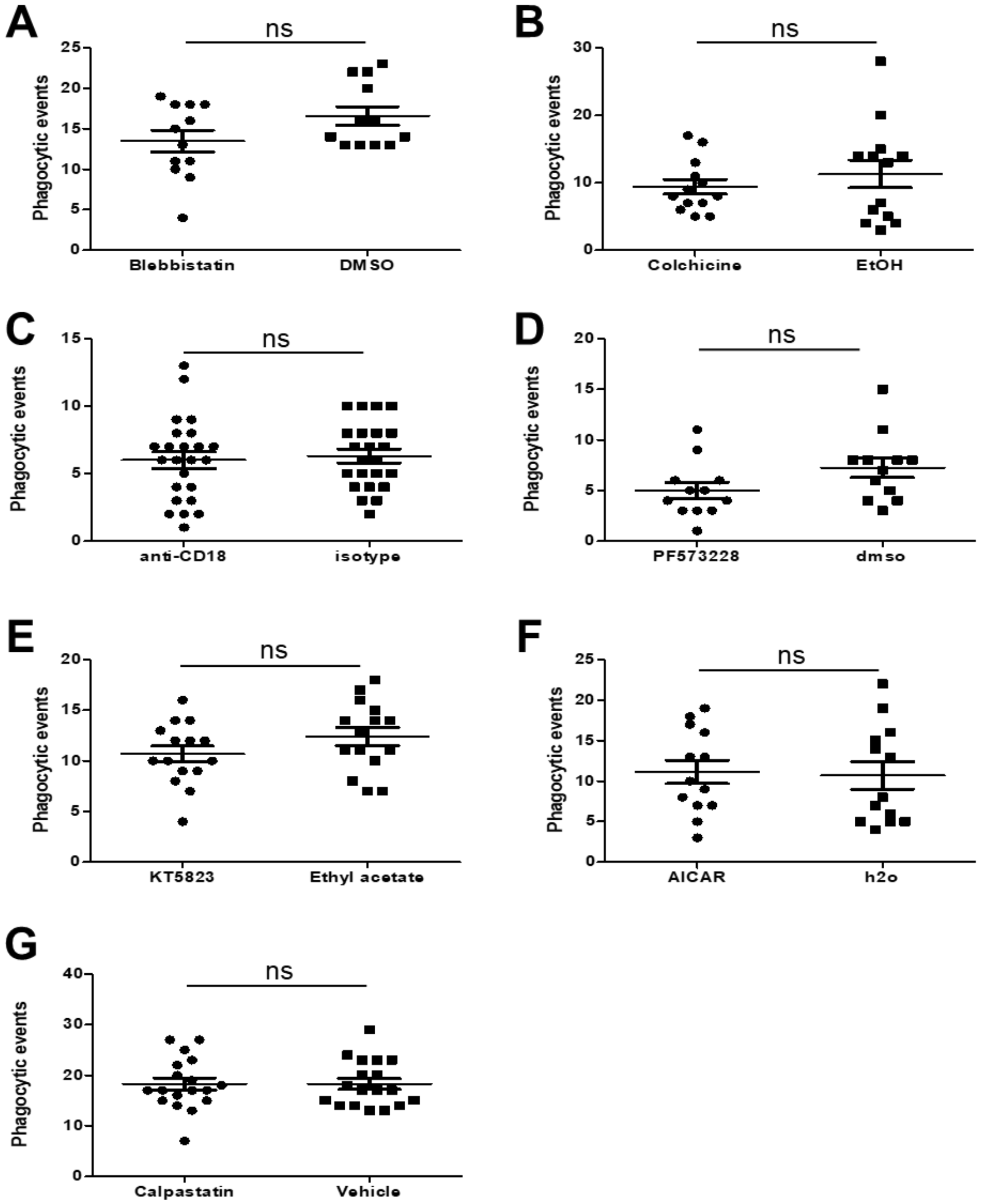

Fig. S1. Impact of treatments upon the phagocytosis of *C. albicans tup1*Δ filaments by macrophages at the concentrations used in the fungal folding assays (above). (A) blebbistatin. (B) colchicine. (C) anti-CD18. (D) PF573228. (E) KT5823. (F) AICAR. (G) calpstatin.

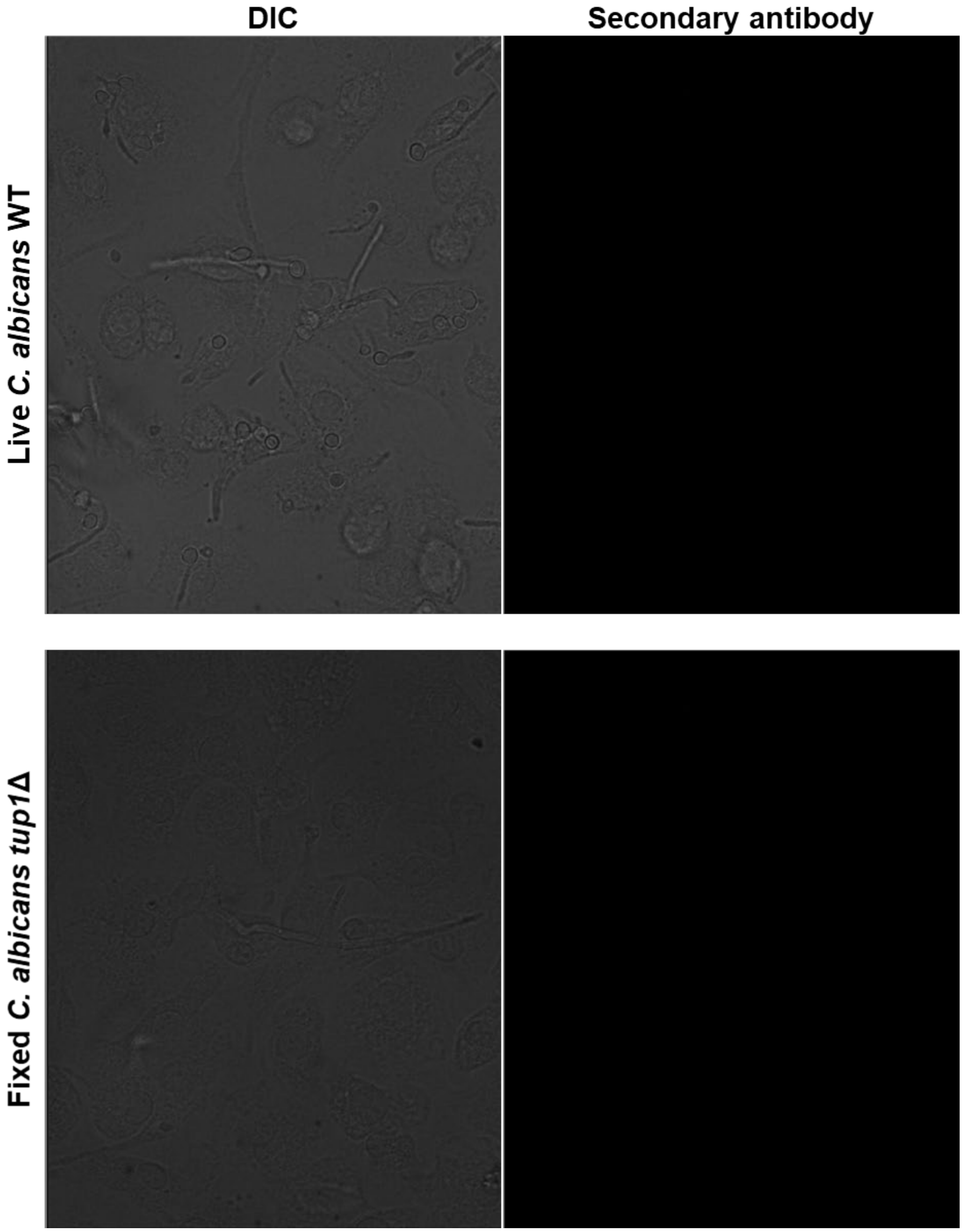

Fig. S2. Secondary antibody controls for localisation experiments. Live *C. albicans* SC5314 or fixed *tup1*Δ cells were mixed with murine BMDMs, fixed after 3 h, stained and subjected to phase differential interference contrast (DIC) and fluorescence microscopy. These same cell populations were used to examine vinculin, paxillin or talin localisation. The same secondary antibody was used to analyse dectin-1, vinculin, paxillin and talin localisation, and no significant background staining was observed for this antibody.

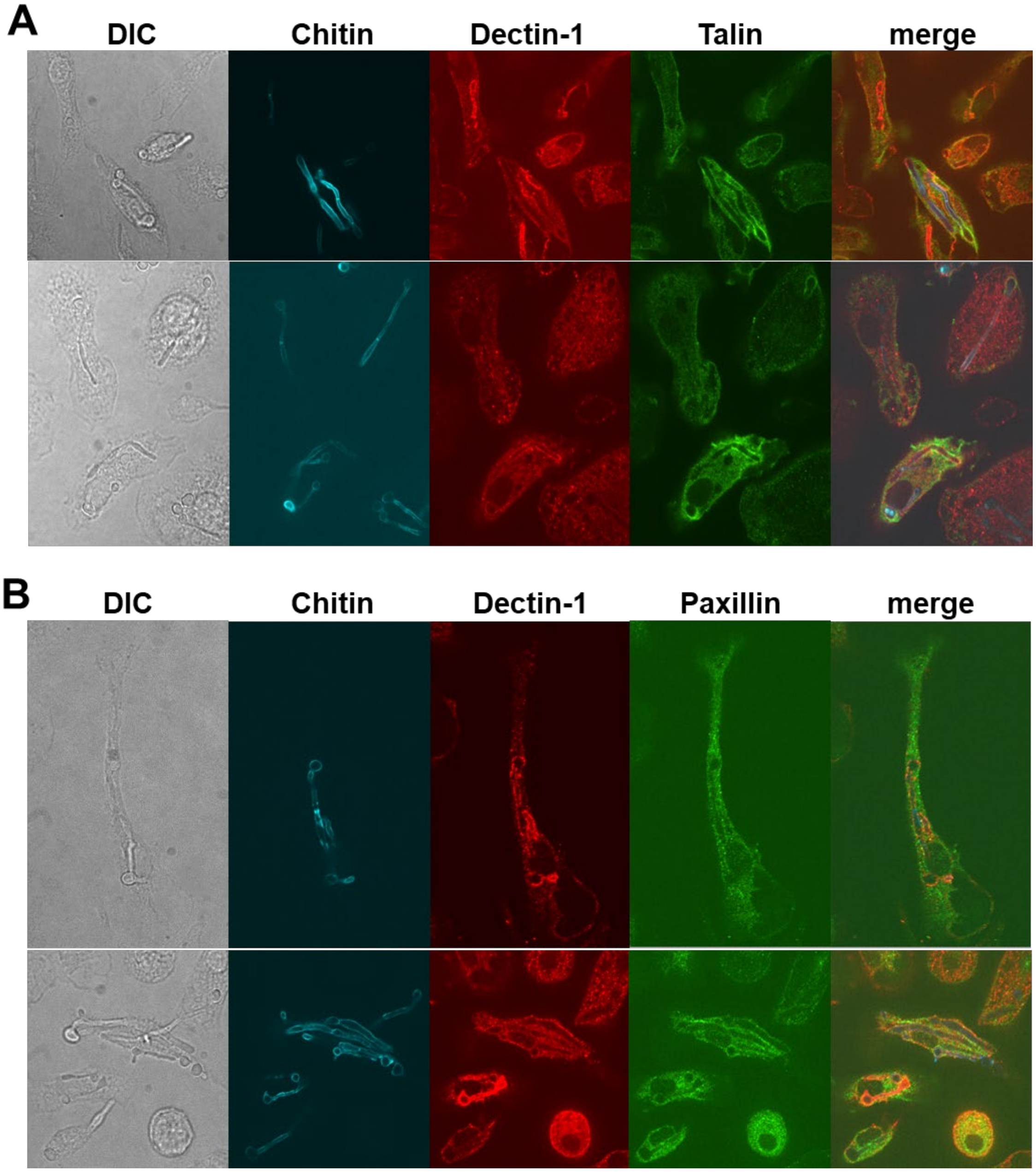

Fig. S3. Juxtaposition of dectin-1, talin and paxillin around phagocytosed *C. albicans* hyphae. Interactions between live *C. albicans* SC5314 and murine BMDMs were monitored. After 4 h, cells were fixed, stained and subjected to phase differential interference contrast (DIC) and fluorescence microscopy: fungal chitin (Calcofluor White; CFW, blue); dectin-1 (anti-dectin-1, red); talin (anti-talin antibody, green (A)); paxillin (anti-paxillin antibody, green (B)). dectin-1 did not appear to co-localize with talin (A) or paxillin (B).

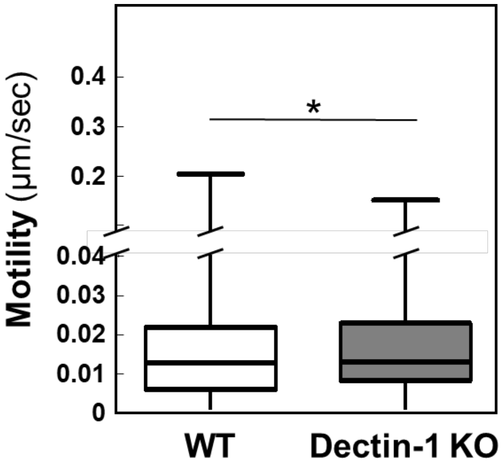

Fig. S4. Motility of dectin-1 knockout macrophages compared to their wild type control. Dectin-1 knock-out (KO) macrophages displayed slightly increased motility (0.0178 ± 0.00028 µm/sec) compared to wild type macrophages (WT: 0.0170 ± 0.00027 µm/sec): $p = 0.0221$.

Movie S1 (separate file). BMDM engulfs and folds a live *C. albicans* SC5314 hypha. This video represents approximately 360 minutes of live imaging of macrophage-*C. albicans* interactions (Materials and Methods). The movie shows multiple folding events and fracture of the hypha that ends up centre stage.

Movie S2 (separate file). Thio-mac containing a live *C. albicans* SC5314 hypha, which is folded. This video represents approximately 240 minutes of live imaging of macrophage-*C. albicans* interactions (Materials and Methods), during which the hypha is folded and damaged.

Movie S3 (separate file). J774.1 cell engulfs a live *C. albicans* SC5314 yeast with germ tube. This video, which represents approximately 200 minutes from a 4 h movie (Materials and Methods), shows the engulfment of a mother cell and short germ tube by a macrophage, the growth of the germ tube in the macrophage, and then the resultant filament being folded by the macrophage.

Movie S4 (separate file). RAW264.7 cell engulfs and folds a live *C. albicans* SC5314. This video represents approximately 360 minutes (Materials and Methods). It shows a macrophage trapping and engulfing a yeast cell that is undergoing morphogenesis. The germ tube continues to extend, and the filament is then folded by the macrophage.

Movie S5 (separate file). Human monocyte-derived macrophages phagocytosing and folding live *C. albicans* SC5314. In this video, which represents 360 minutes in real time (Materials and Methods), the macrophage centre stage phagocytoses two *C. albicans* yeast cells, which undergo morphogenesis. The germ tubes continue to extend, and then both hyphae are folded. Meanwhile, the macrophage below it (and to the left) folds another hypha twice.

Movie S6 (separate file). BMDM engulfs a long fixed *C. albicans tup1*Δ hypha. In this video, which represents about 93 minutes in real time (Materials and Methods), a macrophage starts to phagocytose a long *tup1*Δ filament and, as it does so, the macrophage folds the filament allowing it to fully engulf the fungus.

Movie S7 (separate file). BMDMs engulf and fold fixed *C. albicans tup1*Δ hyphae. This video represents 200 minutes in real time (Materials and Methods). It shows two macrophages (middle left) that trap and phagocytose *tup1*Δ hyphae. Then a third macrophage is shown folding another hypha multiple times (bottom).

Movie S8 (separate file). BMDM engulfs and folds a hyphal filament of the fungal species, *M. sterilia*. This video, which represents 60 minutes in real time (Materials and Methods), shows a macrophages folding a *M. sterilia* hypha, revealing that macrophages can fold the hyphae of an evolutionarily divergent, non-pathogenic environmental fungus.

Movie S9 (separate file). BMDM engulfs and fragments a short *C. albicans tup1*Δ hypha. This video represents 200 minutes in real time (Materials and Methods). The macrophage (starting centre stage) engulfs a relatively short fixed *C. albicans tup1*Δ filament. The macrophage then folds the filament at the yeast-hypha junction, and severs the yeast part from the remainder of the filament.

Movie S10 (separate file). Live 3D imaging of macrophage actin dynamics over a *C. albicans* filament. This video represents 107 minutes in real time (Materials and Methods). A fixed *tup1*Δ hypha stained with CFW (blue) is phagocytosed by a BMDM, and the actin patterns are shown to change dynamically over the dead fungal filament using silicon rhodamine live actin staining (orange).

Movie S11 (separate file). Live 3D imaging of macrophage actin dynamics over an emerging *C. albicans* germ tube. This video represents 103 minutes in real time (Materials and Methods). A RAW264.7 macrophage expressing Life-ACT (green) displays intense and dynamic actin patterns around phagosomes as live *C. albicans* SC5314 cells are engulfed. Dynamic actin persists around phagosomes formed for some time, around a hypha emerging from a mother yeast cell.
